# Limitations in SELDI-TOF MS whole serum proteomic profiling with IMAC surface to specifically detect colorectal cancer

**DOI:** 10.1186/1471-2407-9-287

**Published:** 2009-08-19

**Authors:** Qi Wang, Jing Shen, Zhen-fu Li, Jian-zheng Jie, Wen-yue Wang, Jin Wang, Zhong-tao Zhang, Zhi-xia Li, Li Yan, Jin Gu

**Affiliations:** 1 Key laboratory of Carcinogenesis and Translational Research (Ministry of Education), Department of Colorectal Surgery, Peking University School of Oncology, Beijing Cancer Hospital & Institute, Beijing, PR China; 2 Central Laboratory of Biochemistry and Molecular Biology, Peking University School of Oncology, Beijing Cancer Hospital & Institute, Beijing, PR China; 3 Department of General Surgery, China-Japan Friendship Hospital, Beijing, PR China; 4 Department of General Surgery, Beijing Friendship Hospital, Beijing, PR China; 5 Department of General Surgery, Beijing Tongren Hospital, Beijing, PR China; 6 Hepatic Surgery Center, Tongji Hospital, Tongji Medical College, Huazhong University of Science and Technology, Wuhan, PR China

## Abstract

**Background:**

Surface enhanced laser desorption and ionization time-of-flight mass spectrometry (SELDI-TOF-MS) analysis on serum samples was reported to be able to detect colorectal cancer (CRC) from normal or control patients. We carried out a validation study of a SELDI-TOF MS approach with IMAC surface sample processing to identify CRC.

**Methods:**

A retrospective cohort of 338 serum samples including 154 CRCs, 67 control cancers and 117 non-cancerous conditions was profiled using SELDI-TOF-MS.

**Results:**

No CRC "specific" classifier was found. However, a classifier consisting of two protein peaks separates cancer from non-cancerous conditions with high accuracy.

**Conclusion:**

In this study, the SELDI-TOF-MS-based protein expression profiling approach did not perform to identify CRC. However, this technique is promising in distinguishing patients with cancer from a non-cancerous population; it may be useful for monitoring recurrence of CRC after treatment.

## Background

Colorectal cancer (CRC) is ranked the fourth most frequent cause in cancer-related deaths in China [1]. Pre-symptomatic screening can detect early-stage cancer while it is still localized and with potential to be cured, translating into greatly reduced cancer-related mortality and treatment-related morbidity. Unfortunately, only about 37% of CRC remain localized at the time of diagnosis [2]. Endoscopic examination of the colon remains the gold standard for diagnosis; however, it is invasive, unpleasant and carries risk of morbidity and even mortality. Identification of high risk patients using simple, cheap and less invasive tests would increase the chance to diagnose CRC at early stage. Carcinoembryonic antigen (CEA) is of proven benefit in prognosis and follow up, but has limited sensitivity (30–40%) for detecting early CRC [3,4], whereas serial faecal occult blood testing is proven to reduce CRC mortality but suffers from significant false negative and false positive rates [5]. Stool DNA analysis of multiple markers has shown to be a potential method for screen detection of CRC [6,7]. However, serum-based assays with equivalent sensitivity and specificity would be more acceptable to many patients. Specifically, methods for early detection or identification of aggressive CRC cancers need to be investigated and developed [8].

Comparative proteomic profiling coupled with computerized machine learning without the need of actual identification of specific proteins recently presented itself as a rapid and promising alternative method to traditionalsingle protein assays. SELDI-TOF-MS is a technology that can produce proteomic "fingerprints" from biological samples using a relatively high throughput platform. SELDI has been applied in identifying diagnostic markers in ovarian [9], prostate [10,11], breast [12], bladder [13], hepatic [14,15] and pancreatic cancer [16] using serum or plasma. Our and several other small case-control studies have also reported that, based on protein profiles of serum, SELDI-TOF MS can be used to separate patients with CRC from healthy controls [17-19]. Once a biomarker for early detection is identified, specific criteria must be met before such a biomarker is accepted as clinically useful following a general process for the identification and validation [20].

Here we describe the results of a validation study of our previously reported [19]markers identified by SELDI-TOF MS for early detection of CRC. This study was designed to determine whether the SELDI-TOF MS method accurately predicts the presence of CRC in an independent, case-control series collected by multiple sites.

## Methods

### Patients and Specimens

Patient samples were enrolled from 4 hospitals in Beijing including Beijing Cancer Hospital, Beijing Tongren Hospital, China-Japan Friendship Hospital and Beijing Friendship Hospital during the period of July 2006 through February 2008. The study was approved by research ethics committees of these hospitals. After obtaining informed consent from patients and volunteers, blood specimens were collected. We used patient information to classify samples into 1 of 4 diagnostic groups: 1) CRC; 2) a control group with a history of non-cancerous disease but no history of any cancer type; 3) a control group without history of CRC, but with other cancer; 4) healthy volunteers. The number of acceptable specimens in each group was 154, 45, 67 and 72, respectively. We selected a random sample of eligible specimens under restrictions imposed to achieve greatest balance by age and sex. The demographic information for all collected samples was provided in Table 1 and contribution of samples by disease groups from each hospital was shown in Table 2. The differences in contributions to disease groups by individual hospital were significant (*P *< 0.0001, Pearson Chi-Square test). As preanalysis sample handling may affect the results [21], all serum was collected following a standard procedure as previously described [19].

**Table 1:** Patient characteristics

	Age		Gender			Clinical Stage			
Sample Group	(mean ± SD)	*P*	Male	Female	*P*	I	II	III	IV
CRC (n = 154)	62.52 ± 11.32		84	70		27	50	69	8
OC (n = 67)	59.16 ± 11.71	0.61*	24	43	0.01†	19	18	15	15
N D (n = 45)	58.66 ± 13.56	0.06*	26	19	0.70†				
HV (n = 72)	50.54 ± 13.94	0.00*	36	36	0.52†				

*Student's *t *test, compared with CRC; † Pearson Chi-Square test, compared with CRC. OC, other cancer types; ND, non-cancerous disease; HV, healthy volunteer.

**Table 2:** Number of selected serum samples contributed by each hospital, by diagnostic group

Hospital	Diagnostic group			
	
	Cancer		Non-cancerous Control	
		
	CRC	OC	Non-cancerous Disease	Healthy Volunteer
		
BCH	67	67	5	0
BTH	24	0	3	4
CJFH	25	0	15	57
BFH	38	0	22	11
Total	154	67	45	72

BCH, Beijing Cancer Hospital; BTH, Beijing Tongren Hospital; CJFH, China-Japan Friendship Hospital; BFH, Beijing Friendship Hospital; OC, other cancer types.

### ProteinChip Processing

On the day of analysis, serum samples were placed on ice and thawed completely. Any samples that were hemolyzed or visually lipemic were excluded from analysis. All samples were subjected to the same number of freeze-thaw cycles.

Because IMAC3 ProteinChip arrays used in our previous study were not commercially available anymore [19], sera were analyzed on Cu2+-loaded IMAC30 ProteinChip arrays (a modified second generation IMAC3) in this study [22]. Each array was run with at least 1 quality control (QC) serum sample, leaving 7 wells for specimen analysis. The QC was a pooled serum sample of 50 volunteers, whom were stringently examined to ensure they do not have hepatitis, renal disease, cancer, inflammatory, malnutrition, or other diseases that might affect body protein metabolism. They were required to take no-meat meals on the day before sample collection, and all the samples were collected in the morning before food intake. To eliminate potential confounding of diagnostic groups with random array effects, each array consisted of randomly selected samples from different diagnostic groups. Sample placement was randomized within arrays to eliminate array position bias. The IMAC30 chip processing procedure was as reported [22].

### SELDI Spectrum Generation

All chips were analyzed in a single Protein Biological System IIc TOF MS (PBS-IIc, Bio-Rad Laboratories) at Beijing Cancer Hospital. The mass spectra were obtained using the following parameters: 175 laser shots/spectra collected in the positive mode; detector sensitivity of 9; and a detector voltage of 2950 V. Mass accuracy was calibrated externally using the All-In-One peptide molecular mass standard (Bio-Rad Laboratories).

### Data Processing

Peaks were detected automatically using ProteinChip Software, version 3.2.1 (Bio-Rad Laboratories). All spectra were compiled and normalized to the total ion currents with baselines subtracted. Peaks between *m/z *1500 and *m/z *50000 were auto-detected using the Biomarker Wizard software (BMW, Bio-Rad Laboratories) with a signal-to-noise ratio of > 5, and the peaks present in > 20% of the spectra were clustered using second-pass peak selection with a signal-to-noise ratio of > 2 and mass windows of 0.3% [23].

### Classifier Construction

A total of 338 specimens were randomly assigned to training set and blinded testing set. Patient characteristics of the two sets were shown in Table 3. There was no statistical difference between the two sets of samples in age (Student's *t *test, *P *> 0.05, detailed data not shown), sex, TNM stage and diagnostic groups from each hospital (Fisher's exact test, *P *> 0.05, detailed data not shown). However, samples of non-cancerous disease from each hospital in the two set were not evenly distributed because of low number of subjects from three participating hospitals (*P *< 0.01, Fisher's exact test). In this study, data were analyzed to develop three individual classifiers for three objectives. The first "Cancer- and CRC-specific" classifier was developed to distinguish patients with CRC from those with other cancer types, and non-cancerous conditions. The second "cancer-specific" classifier was intended to differentiate cancer patients from non-cancerous controls, and the last "CRC-specific" classifier was to distinguish patients with CRC from those with other cancer types.

**Table 3:** Patient characters of the training set and test set

	Training Set				Blinded Test Set			
	
	CRC (n = 78)	Control (n = 89)			CRC (n = 76)	Control (n = 95)		
				
		OC	ND	HV		OC	ND	HV
		33	19	37		34	26	35
Gender								
Male	41	13	10	17	43	11	16	19
Female	37	20	9	20	33	23	10	16
Clinical Stage								
I	13	8			14	11		
II	26	8			24	10		
III	35	9			34	6		
IV	4	8			4	7		
Hospital								
BCH	35	33	4	0	32	34	1	0
BTH	9	0	3	1	15	0	0	3
CJFH	11	0	9	32	14	0	6	25
BFH	23	0	3	4	15	0	19	7

OC, other cancer types; ND, non-cancerous disease; HV, healthy volunteer. BCH, Beijing Cancer Hospital; BTH, Beijing Tongren Hospital; CJFH, China-Japan Friendship Hospital; BFH, Beijing Friendship Hospital.

The Biomarker Pattern Software version 5.02 (BPS, Bio-Rad Laboratories) was used to analyze the proteomic features of the training set data. This software has the ability to combine multiple biomarkers to identify and distinguish any independent groups, thereby increasing sensitivity and specificity compared with single biomarker predictors [24]. A decision tree was generated by using the Gini method with non-linear combinations [23]. Multiple trees were initially generated from 167 samples by adjusting the splitting factor with increments of 0.1. A 10-fold cross-validation analysis was carried out as an initial evaluation of the test error of these trees [24]. The peaks forming the main splitters of the tree with the highest predictive rates were selected, and the tree was rebuilt based on these peaks alone and evaluated by the test set. *P*-values were calculated on the basis of *t*-test (BMW software). A *P*-value of less than 0.05 was considered statistically significant. The validity and accuracy of the best tree was then challenged by the blinded testing set. The samples in the blinded testing set were arranged randomly and their origins were unknown to the technicians who processed them. The same method was used to construct the second decision tree classifier to distinguish cancer patients from non-cancerous controls. Using the cancer specimens in the training set; we then tried to construct the third decision tree classifier to distinguish CRC from the other two cancer types.

### Statistical analysis

Statistically significant differences were detected using the Pearson Chi-Square test and the Student's *t *test. Analysis was performed with SPSS 13.0 for Windows (SPSS Inc). Receiver operator characteristics (ROC) curves were generated and the area under the curve (AUC) values was calculated using this same software.

## Results

### Assay Reproducibility

The reproducibility of the assay was estimated by using QC samples. 18 protein peaks in the molecular weight range from *m/z *1,500 to *m/z *50,000, and the intensity range of 10–60 were selected randomly to calculate the intra-array and inter-array CV. The intra-array and inter-array CV for mass accuracy were both 0.03%, and for the normalized intensity were ≤ 10% and ≤ 20%, respectively (data not shown).

### The Classifier Derived from Previous Study Failed to Discriminate CRC Patients from Control Conditions

**Figure 1:**
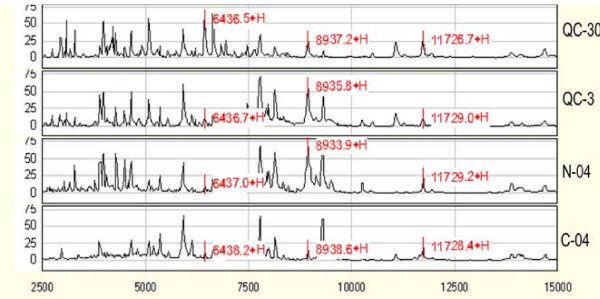
**Raw peak spectra generated by two generations IMAC arrays**. QC-30 and QC-3, a same QC sample on IMAC 30 and IMAC 3 in year 2008, respectively; N-04 and C-04, a normal and a cancer sample on IMAC 3 in year 2004.

We previously reported a classifier (composed of two peaks: *m/z *8,132 and *m/z *4,002) to discriminate CRC patients from healthy volunteers [19]. However, this classifier failed to discriminate CRC patients from healthy volunteers in cohorts of the current study. We then seek to explore reasons for this discrepancy. We used QC samples to generate peaks from an IMAC30 array and a previously preserved IMAC3 array respectively, with the same experimental procedure mentioned above. The *m*/*z *drift between the two arrays was less than 0.03%; but the intensities of peaks generated from the two arrays were of great difference (Figure 1), which might be part of the reasons for the inconsistency. Moreover, we also found that the *m*/*z *drift in the IMAC3 array through time was less than 0.1%, which manifested the reproducibility of this technique.

### SELDI-TOF MS Failed to Discriminate CRC Patients from Control Conditions

To identify serum proteins and polypeptides that are significantly different between CRC and the control serum samples, we carried out peak detection with BMW software after normalizing peak intensities to the total ion current. Seventeen out of 92 peaks from CRC patients were selected, which could be used to distinguish these samples from those from non-cancerous controls in the training and test sets.

**Figure 2:**
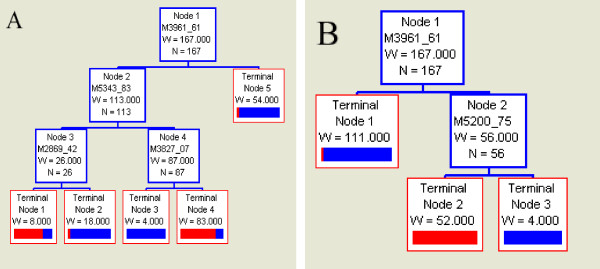
**Decision tree algorithm**. N, the number of specimens; M, the molecular weight. (A) Decision tree analysis to identify CRC and control specimens. If the peak intensity of an analyzed sample is below the cut-off value at the *m*/*z *in the node, the sample proceeds to the left. If not, it proceeds to the right. The cut off value of the peaks were 23.57, 17.42, 3.31 and 0.39, respectively. Red bar, CRC; Blue bar, non-cancerous control. (B) Classification of cancer vs. non-cancerous control specimens by the decision tree algorithm. The cut off value of the peaks were 21.745 and 14.969, respectively. Red bar, cancer; Blue bar, non-cancerous control.

The BPS classification algorithm identified a series of classification models which were constructed with one or more protein peaks with varying classification accuracy. The best classification tree with the highest classification accuracy was constructed using four masses at *m*/*z *3961, 5343, 2869 and 3827 to generate five terminal nodes (Figure 2A). The four peaks achieved a sensitivity of 91.03% and a specificity of 73.03% in diagnosing CRC in the training set.

The best decision tree was then used to predict the presence or absence of CRC in serum samples in the blinded test set. Of the 76 cases of CRC, 16 were misclassified by the decision tree. And among the 95 controls, 4 of the 61 non-cancerous controls, 24 of the 34 patients with other cancer types were misjudged as CRC, respectively. The sensitivity and specificity of the decision tree classification were thus 78.95% (60/76) and 70.53% (67/95), respectively. But 70.59% (24/34) of the control cancers were misjudged; this decision tree thus failed to separate CRC patients from control cancers.

### SELDI-TOF MS Discriminates Cancer Patients from Non-cancerous Controls

**Figure 3:**
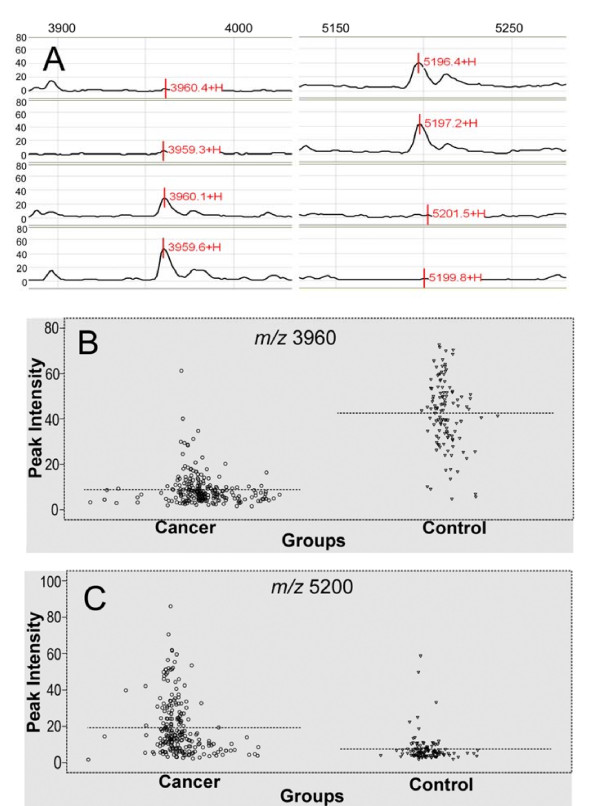
**Detection of two proteins in the mass pattern of serum**. (A) Representative mass spectra showing the two peaks of the classifier at *m*/*z *3961 and *m*/*z *5200. The top two spectra were cancer, and the bottom two spectra were non-cancerous control. (B, C) Cluster plots generated using IMAC 30 ProteinChip for the two peaks of interest. Dotted line, mean peak intensity.

**Figure 4:**
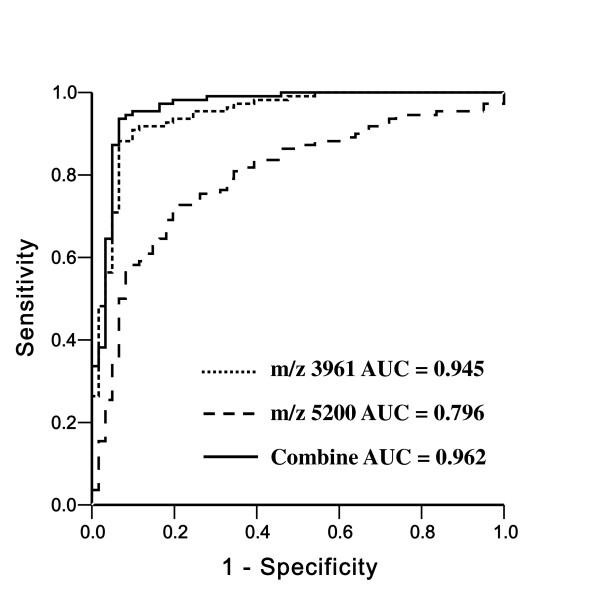
**ROC curves and AUC values**. ROC curves and AUC values showing the discriminating capacities of the *m*/*z *3961 and *m*/*z *5200 peaks individually and combination of two peaks (Combine).

To investigate if SELDI-TOF MS can distinguish cancer patients from a non-cancerous control, the second training set was used to construct a diagnostic serum protein pattern. The best classification tree with the highest classification accuracy was constructed using two masses at *m*/*z *3961 and *m*/*z *5200 to generate three terminal nodes (Figure 2B). The two peaks achieved a sensitivity of 98.20% and a specificity of 89.29% in diagnosing cancer in the training set. Their mass spectra were shown in Figure 3A. These peaks showed significantly different intensity levels between cancer and non-cancerous controls. In the blinded testing set, the sensitivity and specificity of the decision tree classification to separate cancer patients from the non-cancerous controls were 96.36% (106/110) and 90.16% (55/61), respectively. The mean intensity of the *m/z *3961 and *m/z *5200 peaks are of great difference in the cancer group and the non-cancer control group (Table 4). Figure 3B, C demonstrates the distribution of the two peaks of each patient group. The ROC and AUC values of each peak and their combination in the 171 cases of the blind test set are shown in Figure 4.

**Table 4:** Different intensities of *m/z *3960 and *m/z *5200 in patients with cancer and non-cancerous control

Peaks	Intensity (mean ± SD)	* *P*
*m*/*z *3960		< 0.0001
Cancer (n = 221)	8.87 ± 6.97	
Non-cancerous Control (n = 117)	42.49 ± 14.89	

*m*/*z *5200		< 0.0001
Cancer (n = 221)	19.27 ± 15.16	
Non-cancerous Control (n = 117)	7.59 ± 7.58	

*Student's *t *test.

### SELDI-TOF MS Failed to Discriminate Patients with CRC from Control Cancers

To investigate if SELDI-TOF MS can distinguish patients with CRC from those with other cancer types, serum specimens in the training set were used to construct a third diagnostic serum protein pattern. The BPS algorithm failed in building a classifier to reliably separate CRC from the control cancers. The best selected tree performed poorly in the blinded test set with a sensitivity of 60.53% and specificity of 58.82%.

## Discussion

Newly discovered biomarkers must be validated before ultimately used in a clinical setting. As a validation benchmark, Pepe et al [20] proposed five phases in development of biomarkers for early detection of cancer: 1) preclinical exploratory studies, 2) clinical assay development for clinical established disease, 3) retrospective longitudinal repository studies, 4) prospective screening studies, and 5) cancer control studies. The validation effort described in this report addresses phase 2 and 3 and reflects the special challenges of using mass spectrometry-based protein profiling as a biomarker for early detection of CRC.

In the design of this study, we considered previous concerns regarding the propensity for bias in multiplex profiling methods [25], the inherent limitations of protein profiling [26], the effects of pre-analytic sample handling [21] and the related concerns of potential bias and generalizability of this platform [27,28].

We carefully designed the current study to avoid biases in sampling and analysis. To minimize possible sampling bias, we collected samples from four different hospitals in Beijing and selected control subjects with cancers other than CRC, with benign tumor or inflammatory diseases, or healthy volunteers. To minimize the preanalytic sample handling effects, we developed a standard sample processing procedure. The specimens were randomly assigned to training set and blinded testing set with possible confounders such as age, sex, TNM stage and diagnostic groups from each hospital balanced in the two sets. However, there were differences in patient contributions from individual hospital in terms of age, sex and disease group; and samples of non-cancerous disease from each hospital in the two sets were not evenly distributed. These differences were due to distinct nature of patient catchment of each hospital, e.g., specialized cancer hospital versus general medicine hospitals, and represent a potential limitation in our study.

In this study, we failed to use our previously discovered classifiers [19] to detect CRC, which may be due to the internal difference between the IMAC 3 ProteinChip and its second generation, IMAC 30, for, we have shown in this report that peaks generated from these two ProteinChip arrays differed greatly in their capacity to capture protein peaks including number and intensities. Moreover, we failed to find a classifier that reliably identifies patients with CRC from the mixed cohort of patients with other cancer types and control subjects or any classifier that was able to distinguish CRC from other cancer types. We were thereby unable to confirm the promising results reported previously [17-19], which could be seen as phase 1 studies for the discovery of specific biomarkers for CRC.

Recently, the Genitourinary Collaboration Group of the American National Cancer Institute Early Detection Research Network reported a failure in using SELDI-TOF MS whole serum proteomic profiling with IMAC surface to specifically detect prostate cancer [29]. Their work gave us a model for biomarker studies. In their initial study, they found this technique could distinguish prostate cancer patients from benign prostate hyperplasia and healthy men [10]. Encouraged by this finding, they conducted a strict validation process. In the first stage of validation, they proved the platform reproducibility of SELDI-TOF MS over time and across laboratories [30]. When they went further to the second and third stages, they were able to identify deficiencies and implement improvements [29,31]. In one of their reports [31], bias in sample collection protocols (i.e., the samples were not collected under a same strict process) were discovered and a uniform procedure in sample preparation was recommended.

Based on these reports, our study design avoided potential biases in sample colleting procedures by using a uniform protocol; we did not have differences in patient ethnical background that may compromise the study; we attempted and succeeded to randomize and evenly distributed patients and subjects among the majority of key characteristics.

One should not conclude from this current study that a particular method does not work or that conclusions from previous studies were wrong. In fact, there is increasing evidence that serum proteomic analysis may ultimately be turned into valuable clinical tools [29]. But the discovery process is multi-staged, often meets with challenges, and requires vigorous validation. We have demonstrated that the SELDI-TOF MS approach with IMAC30 described in this study has limited diagnostic value for CRC. We suggest that greater attention be paid to choices of specimens used in evaluating merit of subsequent studies. Meticulous study design including sample cohort construction is essential and may influence study conclusions. We also suggest that all previous and forthcoming biomarkers should be subjected to equally extensive and rigorous validation. This statement calls into question how to accommodate many previous biomarker discovery efforts with hard to obtain "ideal" specimens. This is a serious challenge for biomarker discovery, as all experimental approaches are subject to false discovery from biased specimens.

However, we were able to generate a classifier consisting of two peaks that distinguishes patients with cancers from non-cancerous controls, with promising diagnosis efficiency. The *m*/*z *3961 peak was down-regulated in the cancer specimens, and the *m*/*z *5200 up-regulated. This classifier separated CRC specimens from non-cancerous controls as effectively as other previously reported classifiers [17-19]. But the two peaks were not CRC-specific; they could not separate CRC from other cancer types. As chance is low for a patient to get two or more types of cancers, this classifier may have potential use in the detection of possible recurrence of CRC after treatment like CEA, but with higher sensitivity and specificity than CEA. Such validation efforts are underway in ongoing studies.

At the moment, we have yet to decode the protein identity associated with these two peaks of interest. Based on this early stage finding, we are in the process of enriching these peaks in an attempt to eventually learn the identity of these peaks. In the meantime we are also using the SELDI platform to further validate the potential prognostic or diagnostic value of these peaks. Once validated, decoding of the two peaks would open up more options for convenient future usage.

## Conclusion

In summary, bias in serum specimens of early studies, differences in study design, and limitations of proteins detected by SELDI-TOF MS in unfractionated serum may explain the inability of this study to identify patients with CRC; meanwhile, the internal difference between the IMAC3 and IMAC30 arrays made it impossible to reproduce and validate our previous findings. However, this technique may have potential use in monitoring the relapse of CRC after treatment, and we are conducting a prospective program for that.

## Competing interests

The authors declare that they have no competing interests.

## Authors' contributions

QW and JS conceived of the study, participated in the study, and drafted manuscript. ZL, JJ, WW, JW, ZZ and ZL participated in the study, enrolled patients and coordinated study activities at their centers. LY and JG conceptualized the study design, oversaw the data collection and analysis, and manuscript draft and review. All authors read and approved the final manuscript.

## Pre-publication history

The pre-publication history for this paper can be accessed here:

http://www.biomedcentral.com/1471-2407/9/287/prepub
